# Health status and health behaviours in neighbourhoods: A comparison of Glasgow, Scotland and Hamilton, Canada

**DOI:** 10.1016/j.healthplace.2009.11.001

**Published:** 2010-03

**Authors:** Kathi Wilson, John Eyles, Anne Ellaway, Sally Macintyre, Laura Macdonald

**Affiliations:** a Department of Geography, University of Toronto Mississauga, 3359 Mississauga Rd. N., Mississauga, Ontario, Canada L5L 1C6; b School of Geography and Earth Sciences, McMaster University, Hamilton, Ontario, Canada; c MRC Social and Public Health Sciences Unit, 4 Lilybank Gardens, Glasgow G12 8RZ, Scotland, UK

**Keywords:** Neighbourhood, Health status, Health behaviours, International comparison, Socioeconomic status, Country context

## Abstract

Health status has been demonstrated to vary by neighbourhood socioeconomic status (SES). However, neighbourhood effects may vary between countries. In this study, neighbourhood variations in health outcomes are compared across four socially contrasting neighbourhoods in Glasgow, Scotland and Hamilton, Ontario Canada. Data came from the 2001 wave of the West of Scotland Twenty-07 Longitudinal Study and a 2000/2001 cross-sectional survey conducted in Hamilton. The results of the comparison point to important variations in the relationship between neighbourhood SES and health. While both cities display a socioeconomic gradient with respect to various measures of health and health behaviours, for some outcome measures the high SES neighbourhoods in Glasgow display distributions similar to those found in the low SES neighbourhoods in Hamilton. Our results suggest that a low SES neighbourhood in one country may not mean the same for health as a low SES neighbourhood in another country. As such, country context may explain the distribution of health status and health behaviours among socially contrasting neighbourhoods, and neighbourhood variations in health may be context specific.

## 1 Introduction

Variations in health status by socioeconomic status (SES) at the local level have been found in many contexts. In general, research has shown that individuals living in high SES neighbourhoods have higher levels of self-reported health, lower incidence of mental health problems and emotional distress than individuals living in low SES neighbourhoods (Balfour and Kaplan, 2002; Mulvaney and Kendrick, 2005; Schlundt et al., 2006; Wen et al., 2006; Xue et al., 2005). Further, research on the links between neighbourhood and health has demonstrated that place has an independent effect on health over and above individual characteristics (Macintyre et al., 2002; Kawachi et al., 1999; for a review see Pickett and Pearl, 2001; Riva et al., 2007). However, most research examining neighbourhood effects on health have been conducted in single cities (Chen et al., 2006; Cox et al., 2007; Zunzunegui et al., 2006). While the link between SES and health status at the local level may vary within and between jurisdictions (Borrell et al., 2004) very little research has been conducted on international comparisons. This represents an important avenue of investigation. Such research could identify the nature of variations between places as well as the reasons for their existence.

In recent work, Stafford et al. (2004) compare neighbourhoods and self-rated health among public sector employees in London and Helsinki. They found that overall only a small proportion of the variations in self-rated health could be explained by neighbourhood variations. However, greater neighbourhood SES effects existed between cities, with London showing higher between neighbourhood differences in health. They attribute these findings to higher levels of spatial segregation in London, pointing to the differences in social policies in the UK and Finland. Similarly, Dragano et al. (2007) examine the relationship between neighbourhood SES and risk factors for cardiovascular disease (CVD) in nine cities in Germany and the Czech Republic. Their work showed that the effects of neighbourhood SES varied by country as well as risk factors. Neighbourhood effects were much more pronounced in Germany than in the Czech Republic while controlling for individual social position. However, van Lenthe et al. (2005), in a study of neighbourhood unemployment and mortality in five European countries and the US, found no evidence that the association between these variables was modified by country context.

In summary, a small but interesting group of studies have examined the link between neighbourhood SES and health in the context of international comparisons. Within this research, a key focus has been on mortality (see van Lenthe et al., 2005; Rodwin and Neuberg, 2005) and a commonality amongst most studies is an examination of neighbourhood effect on one health outcome (but see Dragano et al., 2007). In this paper we seek to contribute to the existing body of research by extending health outcome beyond mortality to focus on both morbidity and health behaviours. In doing so, we examine the relationship between neighbourhood SES and health status/health behaviours in two cities in two countries. The research is guided by the following research question: Do neighbourhood effects on health vary in two different countries? To address the research question, and taking advantage of data opportunities, we have selected Glasgow, Scotland and Hamilton, Canada as the basis for our international comparison.

## 2 Background: Glasgow, Scotland and Hamilton, Canada

Glasgow is Scotland's largest city (population of c.650,000) and has a history stretching back to the Stone Age, with the University of Glasgow being founded in 1451. Hamilton (population of c.505,000) was one of the first major towns developed in Canada and was given officially city status in 1846 (Gentilcore, 1987). We have selected Glasgow and Hamilton for this international comparison because of the availability of roughly similar neighbourhood-health data sets collected during the same time period. In addition, there are some strong connections between the two cities and key similarities between Scotland and Canada. For example, large numbers of Scots have emigrated to Canada and Hamilton is sometimes referred to as the ‘most Scottish of Canadian cities’. Over a quarter of the names of the 169 communities and neighbourhoods that have been identified to date in Hamilton, 49 (29.0%) can be found in Scotland or are based on Scottish family names or Scottish words (Kendall, 2006). In the 19th century, the city of Hamilton provided a network of Scottish banks, insurance companies and tradesmen to service the needs of its hinterland of lowland farming communities (Harper, 1996). In addition, Glasgow and Hamilton have strong industrial roots with blue-collar working class populations relying on shipbuilding and steel manufacturing, respectively. As postindustrial cities both have become important service centers with significant educational and health care functions.

Given the focus of the paper we concentrate on health status and health behaviours, which differ between the two countries. In Canada, approximately 11% of the population self-assess their health status as fair or poor (Canada, 2003) and almost one-quarter of the population is obese (Tjepkema, 2005). Approximately 17% of the Canadian population smoke daily, 10% are not physically active and 30% have been hospitalized for at least one night in the past year (Canada, 2005). In Scotland, 26% of the population rate their health as fair or poor and a similar proportion of the population are obese. Almost a third (31%) of Scots are current smokers and 17% are not physically active. Compared to Canadians, a much lower proportion (11%) of the Scottish population have been hospitalized for one night or more in the past year (Bromley et al., 2003).

In this paper we seek to establish the relative importance of neighbourhood SES for health in these two rather similar cities to examine the extent to which living in more affluent or deprived neighbourhoods are similarly associated with health. To explore this question, we utilise data from existing community studies in the two cities.

## 3 Data and methods

Data for this research came from two existing surveys administered to random samples of households in four socially contrasting neighbourhoods in both cities. In both the Glasgow and Hamilton studies cluster analysis and a range of different socioeconomic indicators from the national censuses were initially used to select high and low SES neighbourhoods as study locations for the surveys. Each survey will be discussed in turn.

### 3.1 Hamilton survey

In the Hamilton study, neighbourhoods were selected through a combination of statistical methods that utilized socioeconomic and demographic data extracted at the census tract level from the 1996 Census of Canada (data was taken from this year as the 2001 census data had not been released at the time of our study) in conjunction with smoking data from a random survey of Canadian adults, including Hamilton (see Manfreda et al., 2001). Principal component analysis, local indicators of spatial association (LISA), and geographical information systems were used to identify neighbourhoods representing clusters of 17 socioeconomic (e.g., unemployment, low income, education, dwelling value) and demographic (e.g., recent immigrants, marital status, gender) determinants of health and related risk factors. This analysis was coupled with key informant interviews, which identified similar areas of interest for study. The selected neighbourhoods displayed various combinations of socioeconomic status and social diversity (e.g., lack or presence of recent immigrants, visible minorities, etc.) (see Luginaah et al., 2001 for detailed information on neighbourhood selection and characteristics). The neighbourhoods selected are referred to as Chedoke-Kirkendall, the Downtown Core, Northeast Industrial and the Southwest Mountain. The Northeast Industrial and Downtown Core represent the lower end of the SES spectrum with the Downtown Core representing the most socio-economically deprived neighbourhood, characterized by much lower levels of income, housing tenure and a higher percentage of non-married households than the Northeast Industrial neighbourhood. Chedoke-Kirkendall and the Southwest Mountain represent the higher end of the SES spectrum. Socioeconomic differences between these two high SES neighbourhoods are small.

The sampling frame for the telephone survey was obtained from tax assessment records provided by City of Hamilton officials. This database provided the names and addresses of potential respondents, and telephone numbers were then sought using the Canada 411 Internet locator service. The Institute for Social Research (ISR) at York University conducted the survey, which was administered to a random sample of approximately 300 selected people, aged 18 years and older, in each neighbourhood between November 2001 and April 2002. Only one survey was completed per household. Individuals with the most recent birthday were selected to complete the survey. The survey was completed by individuals aged 18 years and older and had an overall response rate of 60% and a refusal rate of 26%. As is common with telephone surveys, our sample has higher levels of socioeconomic status (i.e., household income, education, housing tenure) than the general population (Grube, 1997; Purdiel et al., 2002). For the Hamilton data, individual weights calculated by the ISR based on the probability of an individual being selected due to differential household size, are used in all statistical analyses.

### 3.2 Glasgow survey

In Glasgow, we draw upon the locality component of the longitudinal ‘Twenty-07 Study: Health in the Community’ study based in the West of Scotland in the United Kingdom (Macintyre et al., 1989). The Twenty-07 study, which began in 1987, is following three cohorts (aged 15, 35 and 55 when first interviewed in 1987) of individuals over a 20 year period. The aim of the West of Scotland study is to explore the social processes which contribute to producing social patterning in health, in particular by sex, age, socio-economic status, ethnicity, family composition and area of residence. In this study we use data from the Glasgow 2001 survey (response rate 63.4%), which constitutes the fourth sweep of the Twenty-07 Longitudinal Study. Data for the 2001 Glasgow survey were collected through the use of face-to-face interviews by trained nurses (Macintyre et al. 2006).

The locality component of the West of Scotland study involves comparing a randomly selected sample of individuals residing in two socially contrasting localities in Glasgow city. In particular, ten post code sectors in two different areas of Glasgow were purposefully selected to capture a range of socioeconomic experiences and environments (Benzeval et al., 2008). The sampling frame used to create samples of specific ages, was Strathclyde Regional Council's Voluntary Population Survey, which is an enhanced electoral register containing information on the age and sex composition of each household. The two localities (each comprising two neighbourhoods) are the North West of the City of Glasgow (comprising the neighbourhoods of the West End and Garscadden); and the South West of the City (comprising the neighbourhoods of Mosspark and Greater Pollok). The two localities were selected from a continuum of eight socio-residential types (based on variables 8 variables (e.g., housing tenure, unemployment, proportion of immigrants)) in the city of Glasgow, the North West locality being towards the ‘better’ pole and the South West locality towards the ‘worse’ pole of this continuum, but not at the extremes (criteria for selection are given in MacIver and Macintyre, 1987). While it is difficult to establish a gradient when dealing with only four neighbourhoods, the West End represents the most affluent neighbourhood in the Glasgow study and Greater Pollok the most deprived. It is though important to note that the differences between the lower SES neighbourhoods are greater in Hamilton than in Glasgow case.

The Glasgow and Hamilton neighbourhood data both come from population health surveys conducted with randomly selected participants. The 2000/2001 Hamilton Survey is a one-time cross-sectional survey (as described above). The Glasgow 2001 survey constitutes the fourth sweep of the Twenty-07 Longitudinal Study, however, we are treating the 2001 Glasgow data as cross-sectional for the purposes of this analysis. The neighbourhood sample sizes ranged from approximately 300 in each of the Hamilton neighbourhoods to a low of 84 and a high of 247 in the Glasgow neighbourhoods. While the sample sizes are large enough to support the data analysis presented in the paper, the small sample in Mosspark (84) requires some caution in interpretations.

### 3.3 Survey participants

In this paper, six variables were selected to represent the socioeconomic and demographic characteristics of the survey participants by neighbourhood in each city (see Table 1). In presenting the results we group together the higher SES neighbourhoods and lower SES neighbourhoods in each city and point to the relative socioeconomic status of all neighbourhoods when reporting health outcomes. For the purposes of this paper, respondent social class is represented by occupation because it was the only SES variable that was available and similarly measured in both surveys. In the Glasgow study, this is based on the occupation of the head of the household, using the British Registrar General's occupation-based classification scheme (OPCS, 1992) using the last known occupation of the head of the respondent's household and divided into six categories (subsequently collapsed into 4 groups—I/II Professional/Intermediate; IInm Skilled/non-manual; IIIm Skilled manual; IV/V Partly skilled/unskilled manual). Occupational social class was classified into the same four groups for the Hamilton survey data.

Since the Glasgow data is longitudinal, at the time of the 2001 survey, the youngest age cohort was aged around 25 years, middle cohort around 45 years and oldest cohort around 65 years. The Hamilton survey data was categorized to closely match the age categories in Glasgow as follows: Youngest Cohort (18–25), Middle Cohort (26–59) and Oldest Cohort (60+). Marital status is represented by four categories including married (and those living with a partner), never married, widowed and individuals who are divorced (or separated). Household size measures the number of people living in each household. Sex is a dichotomous variable coded ‘male’ and ‘female’.

In brief, there is a similar distribution of male and female participants in both surveys across all neighbourhoods (Table 1). There is a higher percentage of Glasgow respondents in the youngest and oldest cohorts and a higher percentage of Hamilton respondents in the middle cohort. In the highest SES neighbourhoods in Hamilton, there is a higher percentage of married respondents and a lower percentage of individuals living alone in the highest SES neighbourhoods as compared to Glasgow neighbourhoods. In both cities, there appears to be a link between neighbourhoods SES and social status. In particular, the percentage of respondents in the highest occupational class is much smaller in the lowest SES neighbourhoods. Interestingly, the percentages are higher in the low SES Hamilton neighbourhoods than in the low SES Glasgow neighbourhoods, suggesting that in general, socioeconomic status may be relatively higher in Hamilton. Further, while the characteristics of survey participants in the low and high SES neighbourhoods in each city are fairly similar, the Downtown Core neighbourhood in Hamilton does stand out in terms of much lower levels of SES relative to the Northeast Industrial. The same cannot be said of differences between Mosspark and Greater Pollok.

### 3.4 Variables and statistical analysis

Seven similar health-related questions were used in both the Glasgow and Hamilton neighbourhood surveys. These questions allow us to compare health and health behaviours between neighbourhoods in both cities. Three health status variables were common to both surveys: self-rated health, emotional distress and chronic conditions. In the Glasgow survey individuals were asked to rate their health on a four-point scale including ‘excellent’, ‘good’, ‘fair’, and ‘poor’. Responses were recoded into a dichotomous variable representing ‘excellent/good’ and ‘fair/poor’. In the Hamilton survey an additional category ranking ‘very good’ was included in the scale. Responses were recoded such that ‘excellent/very good/good’ formed one category and ‘fair/poor’ formed the other category. Emotional distress was measured in both surveys using the General Health Questionnaire (GHQ) (Goldberg and Williams, 1988). The GHQ is comprised of a series of questions asking respondents if they had felt a certain way in the past 2 weeks (e.g., constantly under strain, feeling unhappy or not). Next, using a four-point scale respondents are asked to rate how usual it was for them to have felt this way. The twelve-item version (GHQ-12) was used in Glasgow while the 20-item version (GHQ-20) was used in Hamilton. For the purposes of this research, the GHQ-20 version used in the Hamilton survey was reduced to the GHQ-12. Summing across all items, the cut-off point for emotional distress in adults is a score of 3 (Goldberg, 1972). Thus the variable was recoded into two groups comprised of those scoring 3 or more and those scoring less than 3. Chronic conditions were reported in a slightly different manner between the two surveys. In the Glasgow survey individuals were asked to self-report all the chronic conditions they have (e.g., asthma). In the Hamilton survey, respondents were also asked about the presence or absence of 13 diagnosed chronic conditions (e.g., diabetes, cancer, asthma, arthritis). In both surveys the data were recoded into three categories ‘no chronic conditions’, ‘1 chronic condition’, and ‘2 or more chronic conditions’.

Four variables common to both surveys were selected to represent health behaviours and health care use: Body Mass Index (BMI), hospitalization, smoking, and physical activity. In both surveys respondents were asked to self-report their height and weight. Body Mass Index (BMI) was derived from these measures of height and weight (weight (kg)/height (m^2^)). This variable was coded into three categories: ‘under/normal weight’ (<25), ‘overweight’ (25.0–29.9) and ‘obese’ (30+). Respondents participating in both surveys were asked to indicate if they spent at least one night in the hospital in the past 12 months. This was coded as ‘yes’ or ‘no’. In both surveys the smoking variable is categorized according to ‘current smokers’ and ‘non-smokers’ (includes former smokers). Physical activity was measured using different questions. In the Hamilton survey respondents were asked to indicate how often they participated in any physical exercise or activities in the past 3 months. In the Glasgow survey respondents were asked how many days in an average month they participate “in any sport or exercise for more than 20 min that makes you out of breath”. For ease of interpretation, for both surveys, the physical activity data was recoded into dichotomous variable representing ‘any physical activity’ vs. ‘no physical activity’.

In the first stage of the statistical analysis we crosstabulated neighbourhood of residence by each of the seven health-related variables using chi-square tests of association (see Table 2 for presentation of aggregate data by neighbourhood). The crosstabulations enable us to examine between-neighbourhood differences in health status and health behaviours in Glasgow and Hamilton. In the second stage, logistic regression analysis was used to examine differences in neighbourhood effects on health. In particular, we ran seven separate logistic regression models for both Glasgow and Hamilton with each health status, health behaviour and health care use variable representing the dependent variable and neighbourhood of residence representing the independent variable. Odds ratios for neighbourhood of residence adjusted for age, sex and occupational social class were calculated to examine the relationship between the seven health-related variables and neighbourhood of residence. It is common practice to control for age and sex when predicting health outcomes. In addition, SES is argued to be a key determinant of health. We use occupational social class as a proxy for SES as it is a common variable in both surveys.

In all logistic regression models the most affluent neighbourhood in both cities was set as the reference category (i.e., the Mountain neighbourhood in Hamilton and the West End neighbourhood in Glasgow) and separate odds ratios were calculated for the other three neighbourhoods in each city.

## 4 Results

### 4.1 Univariate analysis

#### 4.1.1 Between-neighbourhood differences in health status and health behaviours

The data for Hamilton reveal that those reporting fair/poor self-assessed health varies from a low of 10% in the highest SES neighbourhoods to over 20% in the lower status neighbourhoods (see Table 2). In Glasgow these figures range from 28% to 38% in the high SES neighbourhoods and 42–45% in the lowest SES neighbourhoods. Differences in the reporting of 1 or more chronic conditions across study neighbourhoods are not significant in either Hamilton or Glasgow. With respect to BMI, differences between neighbourhoods are statistically significant in Hamilton but not in Glasgow, although the patterns of obesity do not follow a SES gradient in either city. There are no significant differences in percentages reporting emotional distress (i.e., GHQ score of 3 or more) in the Glasgow study neighbourhoods but in the Hamilton neighbourhoods the results show higher proportions with such distress in the lower SES neighbourhoods. Differences in hospitalization are significant in Glasgow with higher rates of hospitalization in the low SES study neighbourhoods (the Hamilton neighbourhoods shows the same pattern but the results are not significant). Smoking rates and physical inactivity levels are higher in the low SES study neighbourhoods in both cities.

The results show quite interesting differences in the relationship between health status, health behaviours and health care use and neighbourhood SES between the neighbourhoods in the two cities. In particular, in the Hamilton neighbourhoods self-reported health and GHQ vary significantly by neighbourhood but not in the Glasgow neighbourhoods. Similarly, hospitalization shows statistically significant variations across the study neighbourhoods in Glasgow but not Hamilton. Only two variables (smoking and physical activity) demonstrate statistically significant differences by the four neighbourhoods in Hamilton and in Glasgow. The results suggest that the link between neighbourhood SES and health varies by city and depends on how health status, health behaviours and health care use are measured. We note again that city variations may be limited to the chosen affluent and deprived study neighbourhoods.

#### 4.1.2 Relative differences in health and health behaviours by SES between cities

The results also reveal interesting relative differences in health and health behaviours by neighbourhood SES between Hamilton and Glasgow. For example, with respect to self-rated health status, there are 1.5–2 times as many individuals reporting fair/poor health in lower as opposed to higher study SES neighbourhoods in both cities (see Table 2). However, in the Glasgow neighbourhoods there is a much higher prevalence of individuals (two or three-fold depending on neighbourhood) reporting fair/poor health than there are in the Hamilton neighbourhoods. Despite the lack of statistical significance for presence of chronic conditions in the study neighbourhoods in both cities we note little variation between neighbourhoods in either city. However, the percentage reporting 2 or more chronic conditions across all neighbourhoods is more than twice as high in Glasgow compared with Hamilton. Emotional distress shows little variation between neighbourhoods in either city but the rates for emotional distress in Glasgow are almost three times greater in the higher SES study neighbourhoods and approximately 50% higher in the low SES study neighbourhoods than in Hamilton. Conversely, obesity rates are similar across neighbourhoods in Glasgow but in Hamilton the rates are mixed. Rates of hospitalization across neighbourhoods are similar in both cities. Rates of smoking are similar in the higher SES neighbourhoods in both cities but are 30% higher in the lower SES neighbourhoods in Glasgow than they are in the low SES neighbourhoods in Hamilton. Overall physical inactivity rates show enormous variations between the cities’ neighbourhoods. While the rates of inactivity are generally higher in the low SES neighbourhoods in both cities, with respect to higher SES study neighbourhoods, there is a ten to twelve-fold difference in physical activity rates between Glasgow and Hamilton. For lower SES neighbourhoods, there is a five to ten-fold difference. Overall, in both the low and high SES study neighbourhoods, there are higher rates of non-activity in Glasgow as compared to Hamilton.

### 4.2 Multivariate analysis

Finally, we use logistic regression analysis to examine differences in neighbourhood effects on health status, health behaviours and health care use. Odds ratios are presented for neighbourhood of residence adjusted for age, sex and occupational social class (see Table 3). In doing so we seek to determine if neighbourhood of residence is an important determinant of health status, health behaviours and health care use after controlling for key individual factors. The results of this analysis show that the neighbourhood effect remains significant for Greater Pollok (the lowest SES neighbourhood in the Glasgow study) for four of the health measures (self-assessed health, weight, smoking and physical activity) (see Table 3). In addition, residents of Mosspark have higher odds of reporting no physical activity while residents of Garscadden have higher odds of being hospitalized even after controlling for age, sex and occupational status. In Hamilton, the results demonstrate that the neighbourhood effect remains for the Downtown neighbourhood (the lowest SES neighbourhood in the Hamilton study) for GHQ, smoking and physical activity, and for GHQ and smoking in the Northeast Industrial neighbourhood (see Table 3). Thus, the results indicate that in both Glasgow and Hamilton, living in a low SES neighbourhood has a negative influence on health over and above the effects of age, sex and social class.

Overall, however, neighbourhood differences in health (self-assessed health, BMI) and health behaviours (smoking and physical activity) between affluent and deprived study areas appear to be much stronger in Glasgow than in Hamilton. Mental health was the only health status variable where much larger neighbourhood differences were observed in Hamilton than in Glasgow where little neighbourhood differences were found. It is also interesting to note that in both cities, when controlling for age, sex and occupational class, no neighbourhood effects are evident for some health measures. Specifically, in both cities, there are no significant neighbourhood effects for chronic conditions. In Hamilton there are also no neighbourhood effects for self-rated health and hospitalization while the same remains true for emotional distress (GHQ) in Glasgow. Similar to the findings of the univariate analysis, these results also suggest that neighbourhood is important for some health measures in some locations (i.e., the effect varies by city).

## 5 Limitations

Before discussing the importance of these findings, a few methodological and interpretive issues deserve mention. Firstly, the neighbourhood structure in Hamilton and Glasgow may differ substantially with respect to housing type, green space, availability and access to health enhancing amenities. Such neighbourhood characteristics may play a role in explaining not only neighbourhood-level health differences between Hamilton and Glasgow but also the variations observed between low and high SES neighbourhoods. However, this type of data was not collected in both surveys and thus could not form part of our analysis. Secondly, individuals may be sorted into lower and higher SES neighbourhoods in different ways in Canada and Scotland with there being more access to public housing in Glasgow than Hamilton. Residing in public sector housing in Scotland has been shown to be independently associated with health even after taking other known correlates of health into account such as sex, age, social class and income (Macintyre et al., 1998). In Scotland, the quality of public sector housing is poorer than owner occupied housing and is associated with poorer health (Ellaway and Macintyre, 1998). Thirdly, as mentioned earlier, there is some difference in neighbourhood sample size between the cities, which may or may not have shaped the study outcomes. However, it is important to note that the data in both cities come from population health surveys with samples large enough to support the analysis presented in this research. In addition, differences in data collection methods (i.e., telephone surveys in Hamilton and face-to-face interviews in Glasgow along with differences in the measurement of some health variables) may explain some of the health variations observed (e.g., in reporting of chronic conditions) but this is difficult to test. Another issue in conducting comparative neighbourhood-health research relates to potential differences in the definitions of neighbourhood. However, in the Hamilton and Glasgow surveys there are actually strong similarities in how neighbourhoods were derived. In the Hamilton survey, neighbourhoods were defined through spatial clustering of specific demographic and socioeconomic features with the input of key decision makers in the city. In Glasgow, neighbourhoods were also defined through spatial clustering but with the additional element of being nested within local community council areas. Thus, we do not believe that differences in neighbourhood definition can account for the variations observed in this study. Yet, it is important to note that had the boundaries been drawn differently in either city, the distributions of health and health behaviours by neighbourhood SES may have been different (see e.g., Flowerdew et al., 2008). Furthermore, we note that the differences between the low SES neighbourhoods in Hamilton are greater than in Glasgow, which may explain some of the results. Finally, how health is defined and interpreted may vary between countries reflecting differences in cultural values and lifestyles.

## 6 Discussion

Our findings contribute to the nascent literature on international comparisons of health at the local level. Similar to the findings of some other international comparisons (Curtis et al., 2006; Dragano et al., 2007; Stafford et al., 2004) our results demonstrate that neighbourhood SES is associated with health in both cities. In their studies, both Stafford et al. (2004) and Rodwin and Neuberg, (2005) show that neighbourhood effects are greater in one city. Our research goes one step further by demonstrating that the relative effect of neighbourhood SES on health varies by health status, health behaviours and health care use in each city. Most of the previous research on international comparisons examines the effect of neighbourhood SES on only one health outcome (but see Dragano et al., 2007). In doing so, research runs the risk of overstating the importance of neighbourhood effects in one city. However, by including different measures of health the complexity of the link between neighbourhood SES and health is revealed. In particular, the findings point to important variations in the relationship between neighbourhood SES and health by city. In doing so, our study supports the claims of Macintyre et al. (2002, p.128) that “a more differentiated picture has tended to emerge, in which rather than there being one single, universal ‘area effect on health’ there appear to be some area effects on some health outcomes, in some population groups, and in some types of areas”. More specifically, in the Glasgow study neighbourhoods, living in a poor neighbourhood is independently associated with an increased likelihood of fair/poor self-rated health and being overweight or obese but the same does not hold true in the four Hamilton neighbourhoods. In the Hamilton study neighbourhoods, there are strong associations for poorer mental health (i.e., emotional distress) but this was not the case in the Glasgow study neighbourhoods. Yet, in both cities living in a low SES neighbourhood is associated with a higher likelihood of smoking and physical inactivity, which are important health behaviours. The results not only suggest that some measures of health are more sensitive to neighbourhood SES but that this varies by country, an important area of future investigation. We must stress again, however, that there may be key neighbourhood factors which influence health, health behaviours and health care use that we have failed to capture (e.g., location of recreational facilities, health facilities, grocery stores) (Farley et al., 2006; Giles-Corti and Donovan, 2006). This also represents an important focus for future research.

The results of this comparative analysis reinforce the importance of neighbourhood SES in shaping health and health behaviours. However, the results reveal that the relative importance of SES depends on how health is measured. But significantly we note that differences in health status and health behaviours also depend on neighbourhood composition and potentially country context. The study neighbourhoods in both cities display a gradient with respect to several of these variables, a not uncommon finding in neighbourhood-health research. Yet, the distributions are strikingly different between the Glasgow and Hamilton neighbourhoods. For some outcome measures the high SES neighbourhoods in Glasgow display a health distribution similar to the low SES neighbourhoods in Hamilton. The finding appears to suggest that in general residents in Hamilton are healthier than residents in Glasgow. However, another possibility is that a low SES neighbourhood in one country does not necessarily mean the same for health status, health behaviours and health service utilization as a low SES neighbourhood in another country. For example, this may depend on how low SES is measured and distributed in cities in different countries.

Furthermore, we suggest that the differences in the relationships between neighbourhood SES and health in socially contrasting neighbourhoods may depend on country context. Thus, the findings of neighbourhood studies conducted in one country cannot necessarily be transferred to help understand the distribution of health status, health behaviours and health care use in other countries. Researchers need to invoke national characteristics to help explain neighbourhood differences by SES. In this case of Glasgow and Hamilton part of the explanation might be due to differences in average income levels, access to employment opportunities, amenities and services to support people in their everyday lives as well as the economics and delivery of health care and social security systems in Canada and Scotland. Indeed, Canada has already been noted as having a more equitable social and economic distribution system than other neighbouring developed nations (Ross et al., 2000). Furthermore, the relative importance of health behaviours (i.e., healthy lifestyles) may vary by country, as might the values and beliefs that support this importance. For example, variations in smoking rates may be explained, in part, by differences in levels of social acceptability as well as the timing and extent of smoking bans implemented in the two countries. The extent to which this may hold true awaits rigorous research using neighbourhood-level data from multiple countries.

## Figures and Tables

**Table 1:** Characteristics of survey participants in Glasgow and Hamilton neighbourhoods.

		**Hamilton neighbourhoods %**				**Glasgow neighbourhoods %**			
Variable	Category	Chedoke-Kirkendall *n*=300	Mountain *n*=301	Northeast Industrial *n*=302	Downtown *n*=300	West End *n*=206	Garscadden *n*=174	Mosspark *n*=84	Greater Pollok *n*=247
Social class^a^	I/II Professional/intermediate	66.0	55.4	31.1	32.5	68.3	35.8	24.1	19.8
	IInm Skilled/non-manual	22.5	59.0	31.6	29.3	17.6	20.8	15.7	14.9
	IIIm Skilled manual	3.7	4.3	18.4	11.4	8.8	25.4	32.5	38.0
	IV/V Partly skilled/unskilled manual	7.9	11.3	18.9	26.8	18.9	17.9	27.7	27.3
Sex	Male.	43.4	46.0	48.7	50.8	47.6	43.7	46.4	42.1
	Female	56.6	54.0	51.3	49.2	52.4	56.3	53.6	57.9
Age^b^	Youngest cohort	9.8	10.1	11.0	18.1	20.9	23.6	33.3	26.7
	Middle cohort	68.9	66.1	71.2	59.3	46.1	31.6	26.2	36.8
	Oldest cohort	21.3	23.7	17.8	22.6	33.0	44.8	40.5	36.4
Marital status^c^	Married	64.8	71.0	62.1	46.0	56.8	58.1	54.8	59.9
	Never married	20.9	15.6	20.1	31.5	28.6	24.4	23.8	20.2
	Widowed	4.9	6.2	6.1	6.5	9.7	11.6	15.5	11.3
	Divorced	9.4	7.2	11.7	16.1	4.9	5.8	6.0	8.5
Household size^c^	Lives alone	16.8	9.6	13.8	31.2	22.3	27.7	26.2	19.4
	Household of two	30.8	19.9	27.6	27.7	38.8	34.1	33.3	37.7
	Household of three or four	42.0	50.6	46.2	28.9	35.4	32.9	36.9	36.4
	Household of five plus	2.6	19.9	12.5	12.3	3.4	5.2	3.6	6.5

a Significant across Glasgow and Hamilton neighbourhoods chi-square *p*<0.001.

b Significant across Glasgow and Hamilton neighbourhoods chi-square *p*<0.05.

c Significant across Hamilton neighbourhoods chi-square *p*<0.001.

**Table 2:** Health status and health behaviours across neighbourhoods in Glasgow and Hamilton.

		**Neighbourhoods in Hamilton**				**Neighbourhoods in Glasgow**			
		*High SES (%)*		*Low SES (%)*		*High SES (%)*		*Low SES (%)*	
Variable	Category	Chedoke-Kirkendall	Mountain	Northeast industrial	Downtown	West end	Garscadden	Mosspark	Greater Pollok
Self-assessed health^a^	Fair/poor	10	10	23	20	29	39	42	45
Chronic conditions	None	46	44	44	45	18	15	19	18
	1	29	30	29	24	25	19	24	18
	2+	25	26	27	31	58	66	57	64
BMI^a^	Underweight/normal.	48	42	31	48	37	31	38	32
	Overweight	37	37	39.	35	41	42.	43	40
	Obese	15	21	30	17	21	27	20	28
GHQ^b^	Score of 3 or more	7	7	11	16	19	21	19	26
Hospitalization^c^	Spent at least 1 night in the hospital in the past year	5	9	11	10	4	14	14	17
Smoking^d^	Current smoker	17	17	29	29	19	28	38	40
Physical activity^d^	Not physically active	5	10	11	16	53	68	81	77

a Significant across Hamilton neighbourhoods chi-square *p*<0.001.

b Significant across Hamilton neighbourhoods chi-square *p*<0.01.

c Significant across Glasgow neighbourhoods chi-square *p*<0.001.

d Significant across Hamilton neighbourhoods chi-square *p*<0.001, Significant across Glasgow neighbourhoods chi-square *p*<0.05.

**Table 3:** Probability, by neighbourhood, of having fair/poor health, any chronic conditions, being overweight/obese, scoring 3+ in the GHQ, being a smoker, and being physically inactive, after controlling for sex, age, and social class

	**Fair/poor self-assessed health** (vs. excellent/good)		**Any chronic conditions** (vs. 0 conditions)		**Overweight/obese BMI** (vs. normal)		**GHQ score of 3+** (vs. score<3)		**Hospital inpatient for 1+nights in past year** (vs. 0 nights)		**Smoker** (vs. non-smoker)		**0 Physically Active days** (vs. >0 days)	
	Odds ratio	95% CI	Odds ratio	95% CI	Odds ratio	95% CI	Odds ratio	95% CI	Odds ratio	95% CI	Odds Ratio	95% CI	Odds Ratio	95% CI
**Hamilton**														
Mountain	1.00		1.00		1.00		1.00		1.00		1.00		1.00	
Chedoke-Kirkendall	0.99	0.48–2.06	0.88	0.57−1.34	0.73	0.46–1.14	1.65	0.84–3.22	0.22**	0.08–0.62	1.23	0.76–1.98	0.53	0.21–1.35
NE Industrial	1.47	0.75–2.88	1.00	0.65−1.54	1.52	0.98–2.37	2.32*	1.21–4.45	0.49	0.22–1.12	2.04**	1.29–3.24	1.19	0.55–2.55
Downtown	1.12	0.51–2.43	0.96	0.58−1.57	0.86	0.51–1.43	2.16*	1.04–4.48	0.50	0.19–1.30	2.04**	1.22–3.41	2.53*	1.18–5.43
**Glasgow**														
West End	1.00		1.00		1.00		1.00		1.00		1.00		1.00	
Garscadden	1.10	0.67–1.83	1.06	0.65−1.72	1.53	0.94–2.50	1.06	0.62–1.80	1.99*	1.01–3.92	1.19	0.70–2.05	1.08	0.62–1.87
Mosspark	0.80	0.36–1.76	1.40	0.65−3.02	1.49	0.70–3.16	0.66	0.26–1.63	1.08	0.36–3.25	1.93	0.89–4.16	2.42*	1.06–5.52
Greater Pollok	1.64*	1.02–2.63	1.13	0.71−1.80	2.22**	1.38–3.58	1.17	0.71–1.92	1.16	0.58–2.33	2.40***	1.47–3.91	2.02**	1.19–3.41

*** *p*<0.001, ** *p*<0.01, * *p*<0.05
